# Hydrogen Bond Strengths in Phosphorylated and Sulfated Amino Acid Residues

**DOI:** 10.1371/journal.pone.0057804

**Published:** 2013-03-05

**Authors:** Chaya Rapp, Hadassa Klerman, Emily Levine, Christopher L. McClendon

**Affiliations:** 1 Department of Chemistry and Biochemistry, Stern College for Women, Yeshiva University, New York, New York, United States of America; 2 Skaggs School of Pharmacy and Pharmaceutical Sciences, University of California San Diego, La Jolla, California, United States of America; Bioinformatics Institute, Singapore

## Abstract

Post-translational modification by the addition of an oxoanion functional group, usually a phosphate group and less commonly a sulfate group, leads to diverse structural and functional consequences in protein systems. Building upon previous studies of the phosphoserine residue (pSer), we address the distinct nature of hydrogen bonding interactions in phosphotyrosine (pTyr) and sulfotyrosine (sTyr) residues. We derive partial charges for these modified residues and then study them in the context of molecular dynamics simulation of model tripeptides and sulfated protein complexes, potentials of mean force for interacting residue pairs, and a survey of the interactions of modified residues among experimental protein structures. Overall, our findings show that for pTyr, bidentate interactions with Arg are particularly dominant, as has been previously demonstrated for pSer. sTyr interactions with Arg are significantly weaker, even as compared to the same interactions made by the Glu residue. Our work sheds light on the distinct nature of these modified tyrosine residues, and provides a physical-chemical foundation for future studies with the goal of understanding their roles in systems of biological interest.

## Introduction

Phosphorylation of a serine, threonine, or tyrosine residue is one of the most commonly occurring post-translational modifications in biological systems. In a previous work [1], we investigated hydrogen bond strengths of phosphorylated residues using methyl phosphate as an analogue for phosphoserine (pSer). These studies showed that bidentate interactions, in which two nitrogen atoms on the Arg side chain form hydrogen bonds with two phosphate oxygen atoms, were most favored. Of the 518 protein kinases in the human genome, 90 are tyrosine kinases [2]; as of yet no systematic structural studies have been conducted specifically on phosphotyrosine (pTyr) hydrogen bonding interactions. Experimentally determined structures often show bidentate interactions with Arg, such the interaction between the c-Cbl tyrosine kinase binding domain and the tyrosine phosphorylated Met receptor [3], and the interactions with the phosphotyrosine recognition domain of src homology domains (SH2) [4]. At the same time, we expect pTyr interactions to differ from pSer interactions due to the longer side chain and aromatic ring of the pTyr residue.

Recently, there has been increased interest in tyrosine sulfation, a less common yet biologically significant modification. This modification has been predicted or observed in the N-terminal extracellular domain of most chemokine receptors [5] and is involved in a broad range of physiological processes such as the entry of the HIV-1 virus into the cell via the sulfated CCR5 receptor [6]. Studies of protein systems have shown that sulfotyrosine residues cannot be replaced by phosphotyrosine residues despite the common oxoanion functional group [7]. To investigate the distinct nature of the sulfotyrosine (sTyr) and pTyr residues, we conducted a systematic study of hydrogen bonding in these residues, and compare these to Glu and pSer, which we have studied extensively in a previous work [1]. Since we expect that some of the differences in bonding between sTyr and pTyr can be attributed to the reduced charge, −1 for sTyr vs. −2 for pTyr, we also studied protonated phophorylated residues which carry a charge of −1 (pSer(−1) and pTyr(−1)). These residues are also likely to have biological relevance since their pKa values are not far from neutral (∼6 for pSer and 5.8–6.1 for pTyr [8]–[9]). Quantum mechanical calculations were used to derive partial charges for all modified residues.

To investigate the strength of hydrogen bonding in modified residues, we conducted explicit solvent molecular dynamics on a series of XXX-Gly-YYY tripeptides in which XXX represents hydrogen bonding donors Lys, Arg or Gln, and YYY represents hydrogen bonding acceptors Glu, pSer, pTyr, or sTyr. Implicit solvent potentials of mean force, in which models of hydrogen bonding residue pairs are held in a fixed orientation as hydrogen bonding distances are varied, were used to supplement these studies. Furthermore, we validate the results of our theoretical studies against experimentally determined structures using a survey of hydrogen bonds among modified residues in the Protein Data Bank (PDB) [10].

To examine hydrogen bonding within the context of a protein system, we conducted molecular dynamics simulation of the sulfated N-terminus of the CXCR4 receptor in complex with stromal cell derived factor SDF-1 (also called CXCL12). The CXCR4-SDF-1 complex has been implicated in cancer cell migration and metastasis, and is a therapeutic target in cancer treatment [11]. While the CXCR4 N-terminus is sulfated at several sites, sulfation at Tyr21 has been shown to be most critical for SDF-1 binding, and the Tyr21 residue and its chemokine binding site are conserved among many chemokine receptors and their respective ligands [11]. Simulations were run on structures of the native complex sulfated at Tyr21, as well structures in which phosphotyrosine was substituted for sulfotyrosine in order to compare the effects of these distinct modifications.

## Materials and Methods

### Partial Charges for Modified Residues

Atomic electrostatic potential charges for pSer(−2), pSer(−1), pTyr(−2), pTyr(−1) and sTyr, were calculated in Jaguar [12] using quantum mechanical calculations on truncated versions of each residue (starting from the C_β_ atom). Charges were generated by fitting the molecular electrostatic potential (ESP) to a set of point charges. Geometry optimization was done in vacuo using density functional theory (DFT), B3LYP level of theory, with the 6–31 G** basis set, followed by a single point energy calculation using Hartree-Fock level of theory, the cc-pVTZ(−f) basis set, and the SCRF implicit solvent model [13]. Dipole moments were calculated in Jaguar using the optimized geometries. A table of partial charges for modified residues is supplied in the supporting information, Tables S1 and S2.

### Molecular Dynamics of Tripeptide Systems

To investigate the strength of hydrogen bonds between the Glu, pSer, pTyr, and sTyr residues, and hydrogen bonding donors Lys, Arg and Gln (used to model interactions with the backbone amide), molecular dynamics simulations were run on eighteen Xxx-Gly-Yyy tripeptides, in which Xxx refers to Arg, Lys or Gln, and Yyy refers to Glu, pSer(−2), pSer(−1), pTyr(−2), pTyr(−1), or sTyr. The Desmond program [14] was used for molecular dynamics using the OPLS-AA 2005 [15]–[16] force field and SPC [17] water model with partial charges for the modified residues taken from the quantum mechanical calculations. The system was set in an orthorhombic box with a 10 Å buffer region on each side and 0.15 M NaCl was added. Each tripeptide was subject to minimization followed by six runs, using different random number seeds, of molecular dynamics for 10 ns with a recording interval of 1 ps, at 300 K and 1 atm. Hydrogen bond analysis was done in VMD [18] using the default criteria for hydrogen bond distances and angles (3.0 Angstroms, 20°), and occupancies are reported as a percentage of frames showing a particular hydrogen bond.

### Potentials of Mean Force

**Figure 1:**
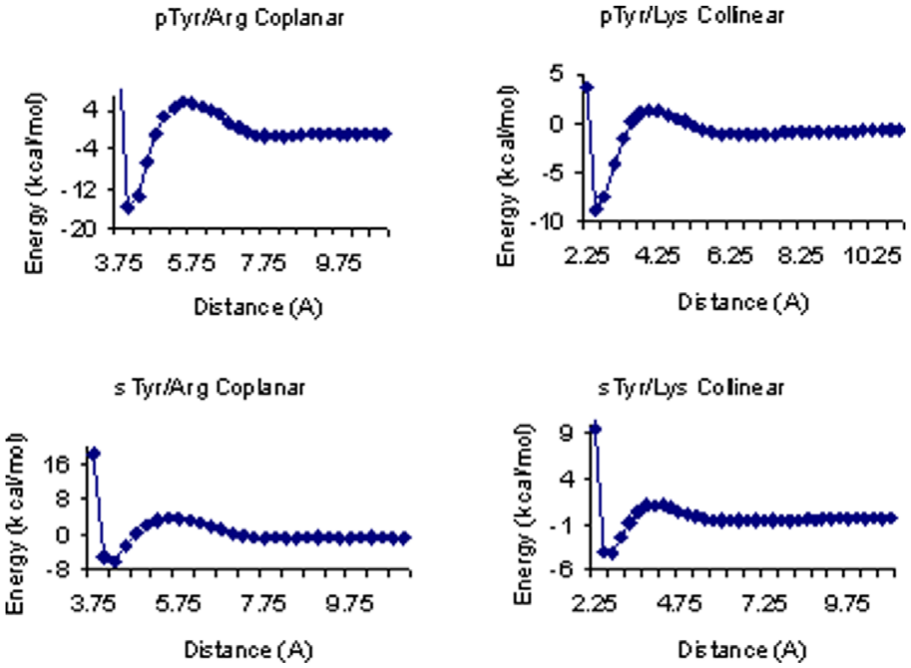
Implicit Solvent Potentials of Mean Force for representative residue pairs. Plots show interaction energy vs. distance, at distance intervals of 0.25 Å, for a pair of residues in a given orientation. Distance refers to the P-C distance between the phosphate atom and the terminal carbon on Arg (coplanar), or the N-O distance (collinear).

Implicit solvent potentials of mean force, on 24 residue pairs, were used to study the strength of hydrogen bonds as a function of hydrogen bond distance and geometry. PMFs were calculated for side chains of Glu (propionic acid), pSer(−2), pSer(−1), pTyr(−2), pTyr(−1), or sTyr, interacting with either a Lys or Arg side chain, or N-methyl acetimide to model interactions with the backbone amide. For interactions with Arg, residue pairs were set up in both a collinear and coplanar bidentate geometry. To construct the residue pairs, we used side chains of each modified residue, taken from the structures generated by the quantum mechanics calculation. Residues were adjusted interactively in Maestro [19] to achieve the desired geometry, and then subject to minimization in Macromodel [20] using a constraint of two 180° angles (N-H-O, H-O-P/S) for the collinear geometries, and a dihedral angle of 0.0° between two Arg hydrogen atoms and two phosphate or sulfate oxygen atoms for the coplanar geometries. Hydrogen bond distances (N-O for collinear, C-C/P/S for coplanar) were varied from 2.25 to 11.0 Å at 0.25 Å intervals, and then at intervals of 0.05 Å, around the region of the minimum. Electrostatic energies were calculated using the Delphi program [21] with partial charges for the modified residues taken from the quantum mechanical calculation, using four grid points per Å, an internal dielectric of 1, an external dielectric of 80, and an ionic strength of zero (altering the ionic strength did not affect the structure of the PMF). These values were added to the Lennard-Jones energies calculated in PRIME [22]–[23]. Interaction energies are reported as the difference between the interaction energy at the minimum and at a distance of 11 Å. Sample PMFs for four systems are shown in Fig. 1.

### PDB Statistics

To conduct a survey of hydrogen bonding among modified residues in experimental protein structures, the PDB was searched for all entries containing modified residues using the three letter amino acid codes SEP, PTR and TYS. The title and resolution of each entry were used to eliminate redundant entries and to keep only the highest resolution structure among entries for the same protein molecule. For hydrogen bonds to the Glu residue, we limited our analysis to the set of PDB entries which were identified to contain a pSer residue. In total, 2126 Glu residues and 136 pSer residues (107 entries), 142 pTyr residues (119 entries), and 13 sTyr residues (10 entries), were studied. PRIME [22]–[23] was used to conduct a crude optimization of each entry and to identify hydrogen bonds based on a heavy atom distance between donor and acceptor atoms that is less than the sum of their atomic radii. This criterion is less rigorous than the distance and geometry criteria used in the analysis of the molecular dynamics trajectories, since hydrogen atom positions are typically not included in PDB entries and can only be inferred. For each residue, we counted the number of hydrogen bonds formed by its side chain. We also characterized hydrogen bonds by donor residue, and for Arg based on whether the interaction is single or bidentate. For Glu, pSer, pTyr and sTyr residues that appear in identical chains in a single entry, we report the number of hydrogen bonds as a rounded average over all chains, and characterize the hydrogen bonds by donor residue for only the first of the identical chains.

### Molecular Dynamics on the CXCR4 Receptor in Complex with SDF-1

Starting structures for molecular dynamics of the CXCR4 N-terminus SDF-1 complex were obtained from the first six conformers of PDB entries 2 k03 and 2 k04. Entry 2 k03 contains conformers of the dimeric complex sulfated at Tyr21 (residues 121 and 321 in chains B and D respectively), while 2 k04 contains conformers of the dimeric complex in its unsulfated form. Phosphorylated structures were prepared from the original 2 k03 conformers by removing the sulfate groups of the sulfotyrosine residues, replacing the residue name ‘TYS’ with ‘PTR’ in the PDB structure file, and building up the phosphotyrosine side chain using side chain prediction in PRIME [22]–[23].

The Desmond program [14] was used for molecular dynamics using the OPLS-AA 2005 force field [15]–[16] and SPC water model [17]. Partial charges for sulfotyrosine were taken from the quantum mechanical calculations described above. The system was set in an orthorhombic box with a 10 Å buffer region on each side and 0.15 M NaCl was added. Minimization was first performed with the solute positions restrained at 50 kcal/mol/Å, for 2000 steps or until forces were below 50 kcal/mol/Å^2^, with 10 steepest-descent steps, then with the solute positions unrestrained another 2000 steps or until forces were below 5 kcal/mol/Å^2^. Then solute heavy atoms were restrained at 50 kcal/mol/Å and NVT molecular dynamics at 10 K was performed for 12 ps using 1 fs time steps for the bonded and short-range nonbonded interactions and 3 fs time steps for the long-range interactions, with the Berendsen [24] thermostat, a relaxation time of 0.1^−1^ ps, and a resampling period of 1 ps. Next, the timestep was increased to 2 fs for the bonded and short range interactions and 6 fs for the long-range nonbonded interactions. NPT equilibration at 10 K for 12 ps using 1 fs time steps for the bonded and short-range nonbonded interactions and 3 fs time steps for the long-range interactions, with the Berendsen [24] thermostat and barostat, using a thermostat relaxation rate of 0.1 ps^−1^, a barostat relaxation rate of 50 ps^−1^, and resampling period of 1 ps. The system was then simulated for another 12 ps at 300 K using the same settings. Finally, heavy atom position restraints were removed and the system is simulated for an additional 24 ps at 300 K with a thermostat relaxation rate of 0.1 ps^−1^ and barostat relaxation rate of 2 ps^−1^.

After minimization and equilibration, production runs of 20 ns were performed on each system using the Martyna-Tobias-Klein integrator [25] at 300 K (Nose-Hoover thermostat [26]) and 1 atm. Snapshots were output every 1 ps. All bonds involving hydrogen atoms were constrained, a 2 fs time step for the bonded and short-range nonbonded interactions was used, and long-range nonbonded interactions were updated every 6 fs using the RESPA multiple time step approach [27]. Short-range coulombic and van der Waals nonbonded interactions were cut off at 9.0 Å, and long-range electrostatics were computed using the smooth particle-mesh Ewald method. Pairlists were constructed using a distance of 10.5 Å and a migration interval of 12 ps.

Hydrogen bonds were identified in VMD [18] using the default criteria for hydrogen bond distances and angles (3.0 Angstroms, 20°), and were averaged over residues in both CXCR4 chains of all conformers. GROMACS [28] was used for calculation of root mean square fluctuation (RMSF) for the backbone of each residue, and averaged for each residue over simulations of all conformers.

## Results and Discussion

### Model Systems and PDB Statistics

**Figure 2:**
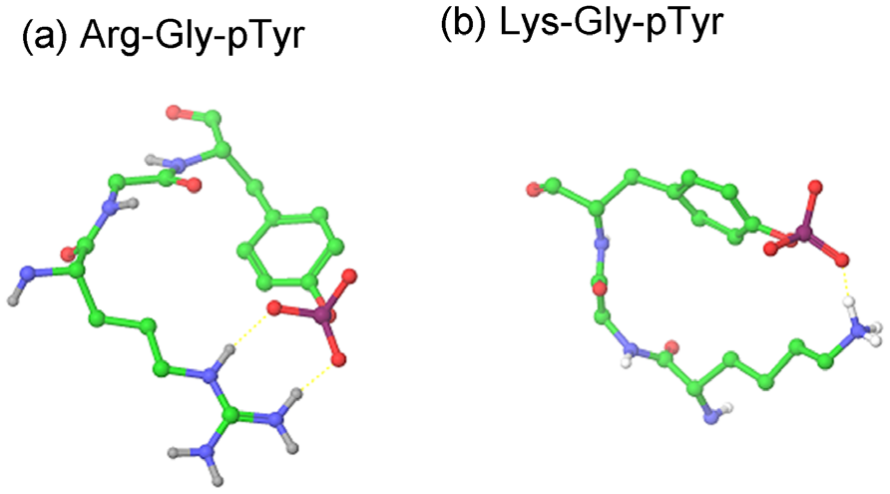
Snapshots from molecular dynamics simulation of two tripeptide systems. Interactions appearing at high frequency are (a) bidentate hydrogen bond in Arg-Gly-pTyr(−2) and (b) single hydrogen bond in Lys-Gly-pTyr(−2). Hydrogen Bonds are indicated by dotted yellow lines.

**Figure 3:**
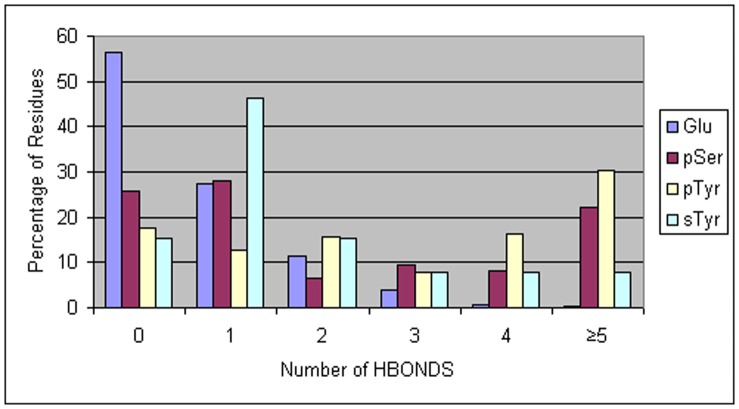
Percentage of Glu, pSer, pTyr and sTyr residues showing a given number of hydrogen bonds. Residues were drawn from all structures in the Protein Databank containing a pSer, pTyr, or sTyr residue. For Glu, residues were taken from the set of structures containing a pSer residue.

The relative strength of hydrogen bonding among Glu, pSer, pTyr, and sTyr residues was assessed based on (1) the frequency of hydrogen bonding between the donor and acceptor residue in the tripeptide simulations (Table 1 and Fig. 2), (2) the depth of the energy minima in the PMFs of residue pairs (Table 2 and Fig. 1), and (3) the overall number of hydrogen bonds (Fig. 3), as well as the frequency of a particular hydrogen bonding interaction (Table 3), observed among residues in structures in the PDB.

**Table 1:** Hydrogen Bond Occupancies in Molecular Dynamics Simulation of Tripeptide Systems.

Acceptor Residue	Hydrogen Bonding Interaction with Donor Residue			
	Arg Single	Arg Bidentate	Lys	Gln
Glu	27.0	21.3	4.4	1.7
pSer(−2)	21.6	66.9	28.4	0.6
pSer(−1)	31.3	7.9	4.2	0.3
pTyr(−2)	27.2	57.7	53.2	0.7
pTyr(−1)	24.3	8.7	16.1	0.4
sTyr	2.5	0.7	0.6	0.3

Columns report the percentage of frames showing a particular hydrogen bond for tripeptides Xxx-Gly-Yyy where Xxx represents hydrogen bonding donors Arg, Lys, or Gln; and Yyy represents Glu, pSer(−2), pSer(−1), pTyr(−2), pTyr(−1), or sTyr. For Arg tripeptides, the table reports percentages for single and bidentate interactions.

**Table 2:** Hydrogen Bonding Energies in kcal/mol computed using Implicit Solvation Molecular Mechanics on residue pairs.

Acceptor Residue	Hydrogen Bonding Interaction with Donor Residue			
	Arg Collinear	Arg Coplanar	Lys	Amide
Glu	−4.1	−11.1	−6.1	−1.5
pSer(−2)	−9.1	−19.7	−10.5	−3.1
pSer(−1)	−5.1	−11.8	−7.1	−2.6
pTyr(−2)	−8.3	−15.6	−8.2	−1.7
pTyr(−1)	−6.7	−11.6	−6.9	−2.7
sTyr	−3.7	−6.0	−4.4	−2.9

Energies are reported as the difference between the interaction energy of the residue pair at the minimum energy and at a distance of 11 Å.

**Table 3:** Characterization of Hydrogen Bonds to Glu, pSer, pTyr, and sTyr in Experimental Protein Structures.

Acceptor Residue	Hydrogen Bonding Interaction with Donor Residue			
	Arg Single	Arg Bidentate	Lys	Amide
Glu	18.5	13.3	13.6	22.7
pSer	12.4	17.8	6.7	26.9
pTyr	23.7	28.6	6.3	18.5
sTyr	4.8	0.0	23.8	33.3

Columns report the percentage of hydrogen bonds to Glu, pSer, pTyr or sTyr, that are to a given donor residue, and for Arg in a single or bidentate orientation. Residues were drawn from all structures in the Protein Databank containing a pSer, pTyr, or sTyr residue. For Glu, residues were taken from the set of structures containing a pSer residue.

For all hydrogen bond acceptor residues studied, our results show that the strongest interactions are with Arg in a bidentate orientation. For the Arg-Gly-pSer(−2) and Arg-Gly-pTyr(−2) tripeptides, 88.5% and 84.9% of frames show hydrogen bonds, and over two thirds of these interactions are bidentate. The implicit solvent PMFs for pSer(−2) and pTyr(−2) show the deepest minima for interactions with Arg in a bidenate orientation at a distance (between the phosphate atom and the terminal carbon on Arg) between 4.00 and 4.05 Å. Bidentate interactions with Arg are also prevalent among experimental structures in the PDB. A breakdown of hydrogen bonding interactions for pSer and pTyr residues in the PDB shows that the highest percentage of interactions are with Arg, and that bidentate interactions are more commonly observed than single interactions.

The frequency of hydrogen bonding to Arg in the tripeptide simulations is reduced by over half for the protonated pSer(−1) and pTyr(−1) residues, and bidentate interactions become less favored than single interactions. The PMFs are shallower for the protonated residues, and there is less of a difference in interaction energies between the coplanar and collinear orientations. These results may explain our finding that for pSer and pTyr residues in the PDB, bidentate interactions with Arg are only moderately favored over single interactions, as both the −2 and −1 states may be stable at physiological pH.

For tripeptides of all residues studied, interactions with the Gln residue are rarely observed (frequency ∼1%). To investigate whether the scarcity of interactions in the Gln tripeptides was due to the shorter side chain of Gln, as compared to Arg and Lys, we conducted two molecular dynamics simulations on tripeptides containing pSer(−2) or pTyr(−2), Gly, and a fictitious residue resembling Gln but with two additional methylene groups inserted in the side chain. We also conducted one 100 ns simulation on the Gln-Gly-pTyr(−2) tripeptide to investigate whether a longer simulation would show a greater frequency of hydrogen bonding. Both the simulations of the altered Gln residue, and the longer simulation, resulted in an almost negligible increase in the observed frequency of hydrogen bonding (data not shown) compared to the original Gln tripeptide simulations. It is unclear why the high frequency of interactions with the backbone amide, observed in the PDB structures for pSer and pTyr, is not reflected in the molecular dynamics simulation of the Gln tripeptides. However, it is possible that in the tripeptide systems, the Gln would lose more conformational entropy through interaction with the phosphate than it would in a protein system where incomplete solvent exposure and the presence of nearby interacting groups would limit its conformational flexibility.

**Figure 4:**
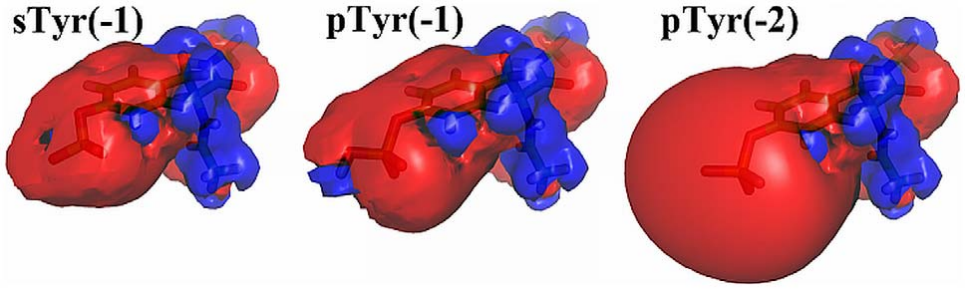
A comparison of electrostatic potentials for sTyr, pTyr(−1), and pTyr(−2). Electrostatic potentials are shown at isosurfaces of +/−2 kTe. The protonated phosphate group of pTyr(−1) presents a shaped charge that can provide a stronger interaction with hydrogen bond donors than the more isotropic charge on the sTyr sulfate.

The sTyr tripeptides show fewer interactions with Arg than any of the other hydrogen bonding acceptor residues, including Glu, and bidentate interactions with Arg are seen in only 0.7% of frames. For the residue pairs involving sTyr, the PMFs show that all minima are notably shallower than minima observed for pSer and pTyr. The overall weaker hydrogen bonding of the sulfotyrosine residue, as compared to the phosphotyrosine, can be attributed to a reduced charge (−1 vs. −2) and a smaller dipole moment (9.4 D for sulfotyrosine and 14.0 D for phosphotyrosine side chains in our Jaguar calculations). Surprisingly, sulfotyrosine hydrogen bonding interactions are also notably weaker than those of the protonated phosphotyrosine residue (pTyr(−1)), which also carries a charge of −1 and has an only slightly higher dipole moment of 9.5 D. To look for a physical rationale for this phenomenon, we visualized and compared electrostatic potentials for sTyr, pTyr(−1), and pTyr(−2) using APBS [29] and PyMOL [30] (Fig. 4), and displayed isovalue surfaces at +/−2 kTe. Obviously pTyr(−2) presents the strongest negative potential, with the negative isosurface extending quite far from the phosphate. In contrast, the differences between sTyr and pTyr(−1) are more subtle. The negative isosurface of sTyr extends in a mostly isotropic manner from the sulfate, whereas in the pTyr(−1) the presence of the proton on one phosphate oxygen presents a shaped charge, with the negative isosurface extending a little farther from the unprotonated oxygen atoms. This shaped charge can be rationalized by resonance Lewis structures, which would have the negative charge shared among three oxygen atoms in sTyr, and two oxygen atoms in pTyr(−1).

The PDB statistics show that pTyr has a strong preference for Arg over interactions with all other hydrogen bonding donors. This preference may be due to cation-π interactions [31] between the positively charged Arg residue and the aromatic ring of tyrosine, which have been shown by others to be stronger for Arg than Lys [32]. One would expect that cation-π interactions would impact the strength and frequency of sTyr interactions with Arg and Lys in the same way. However, we note that the contribution of these interactions is not necessarily represented in typical molecular mechanics force fields, and may require quantum mechanical models or polarizable force fields.

To compare the statistical propensity of acceptor residues Glu, pSer, pTyr and sTyr to form hydrogen bonds, we counted the number of hydrogen bonds formed by the side chains of each of these residues in structures in the PDB. Results show that pSer and pTyr residues have a similar tendency to form hydrogen bonds; 20–30% of residues show five or more hydrogen bonds to surrounding residues. Multiple hydrogen bonds result from each acceptor oxygen atom on the modified residue interacting with more than one donor residue, or more than one atom on a donor residue. For sTyr, the small number of residues studied (13 residues in 10 PDB entries) precludes any statement regarding general trends.

We note that PMFs are useful as an indicator of general trends rather than for their quantitative value, since the residues in each interacting pair are held rigid and there is no contribution from conformational entropy. Furthermore, though our survey of experimental structures is limited to the set of structures currently included in the PDB, our data set is large enough to provide information for all acceptor residues with the exception of sTyr.

Our results regarding hydrogen bonding in phosphorylated and sulfated residues can be verified experimentally by multidimensional NMR experiments on the tripeptide systems that were modeled by molecular dynamics simulation, using ^15^N, ^13^C, and possibly ^33^S or ^77^Se (as a sulfur analogue) [33]. The strength, length and orientation of a particular hydrogen bond are reflected in spin-spin coupling constants, ^2^J or ^3^J, that can be measured by NMR experiments [34]–[36]. Additionally, results of experiments using different solution pH values can be used to characterize how protonation of the phosphate group affects hydrogen bonding in phosphorylated systems. Theoretical studies, using density functional theory for example, can be used to complement the results of these experiments with quantum mechanical calculations of NMR hydrogen bond parameters such as spin-spin coupling constants [37].

### Molecular Dynamics on the CXCR4 Receptor in Complex with SDF-1

To compare interactions involving sulfotyrosine and phosphotyrosine residues in a protein context, we performed molecular dynamics simulations on the CXCR4 receptor in complex with SDF-1 (PDB 2 k03), with Tyr21 either unsulfated (PDB 2 k04), sulfated (PDB 2 k03), or phosphorylated (PDB 2 k03 modified). The simulation of unsulfated CXCR4 N-terminus in complex with SDF-1 showed hydrogen bonds between unsulfated Tyr21 and Glu15 in 4.9% of frames. The simulation of the sulfated complex showed hydrogen bonds between sulfated Tyr21 and both Asn46 and Arg47, in 10.7% and 7.6% of frames respectively. The significance of these residues has been highlighted by structural and binding studies. Arg46 and Arg47 comprise part of the “40’s loop” of SDF-1, which together with the “N loop” forms a basic pocket which binds the sulfotyrosine residue [11]. Furthermore, Arg47 has been shown in binding studies to be critical for CXCR4 N-terminus SDF-1 binding; substitution of Arg 47 by either a neutral Ala residue or a negatively charged Glu residue results in 3.9 and 181.7 fold reductions in CXCR4 activation respectively [11].

The simulations with the Tyr21 residues phosphorylated instead of sulfated showed more frequent interactions with Arg47 (40.5% of frames), and a greater occurrence of bidentate interactions than the sulfated structures (21.7% of hydrogen bonding frames vs. only 0.5% of hydrogen bonding frames). The phosphorylated simulations also show interactions with Asn46 (4.5% of frames) and other residues which are not observed to hydrogen bond to any significant extent in the sulfated simulation: His17 (25.4%), Lys43 (3.4%), and Lys138 (7.2%). An overall higher frequency of hydrogen bonding in the phosphorylated system is consistent with the greater frequency of hydrogen bonding observed for pTyr in the tripeptide simulations, and the more favorable interaction energies in the pTyr PMFs.

A comparison of the sulfated and phosphorylated simulations shows reduced flexibility in the phosphorylated constructs, as reflected by lower values of root mean square fluctuation (RMSF) for certain segments of SDF-1 (the N loop, the loop between beta sheets one and two, and the loop between beta sheet 3 and the helix). For the N loop (residues 11–23), average RMSF values, averaged over six simulations, are 0.82 Å and 0.67 Å, for the sulfated and phosphorylated structures respectively. In the two step model of chemokine activation, the SDF-1 core binds the CXCR4 N-terminus while the flexible SDF-1 N loop controls receptor signaling [38]. The greater flexibility of the sulfated system, which may be attributed to weaker hydrogen bonding interactions, appears to be a distinct and biologically relevant feature of this modification.

### Conclusion

Our preliminary study of sTyr shows that sTyr interactions differ significantly from those of pTyr, consistent with a distinct role for sulfation in biological systems. Molecular dynamics simulations in explicit solvent show notably reduced frequency of hydrogen bonding interactions, as compared to pTyr in either the −2 or −1 protonation states. These results are supported by shallower minima in the implicit solvent PMFs, and more limited hydrogen bonding among the few experimental structures studied. The distinct hydrogen bonding patterns for sTyr and pTyr suggest that the sulfate group is responsible for the distinct nature of the sTyr residue, rather than the properties of the tyrosine residue. More studies will be necessary to further elucidate the physiochemical properties of the sTyr residue, and the structural and functional consequences of tyrosine sulfation in different protein systems.

## Supporting Information

Table S1 **Partial Charges for pTyr(−2), pTyr(−1) and sTyr.**
(DOC)

Table S2 **Partial Charges for pSer(−2) and pSer(−1).**
(DOC)
