# Supplementary material for: Hydrogen Bond Strengths in Phosphorylated and Sulfated Amino Acid Residues
Source: PLoS One. 2013 Mar 5;8(3):e57804. doi: 10.1371/journal.pone.0057804 (PMC3589483; doi:10.1371/journal.pone.0057804)
Supplement: Table S1 — Partial Charges for pTyr(−2), pTyr(−1) and sTyr. (DOC) [file pone.0057804.s001.doc]

Table S1: Partial Charges for pTyr(-2), pTyr(-1) and sTyr.

| *pTyr(-2)* | | *pTyr(-1)* | | *sTyr(-1)* | |
| --- | --- | --- | --- | --- | --- |
| Atoms | Charges | Atoms | Charges | Atoms | Charges |
| CB | -0.4601 | CB | -0.5065 | CB | -0.4776 |
| HB1,HB2 | 0.1880 | HB1,HB2 | 0.2141 | HB1,HB2 | 0.2055 |
| CG | 0.1419 | CG | 0.2476 | CG | 0.2295 |
| CD1,CD2 | -0.1892 | CD1,CD2 | -0.2440 | CD1,CD2 | -0.2484 |
| HD1,HD2 | 0.1736 | HD1,HD2 | 0.1893 | HD1,HD2 | 0.1942 |
| CE1,CE2 | -0.5264 | CE1,CE2 | -0.4159 | CE1,CE2 | -0.3612 |
| HE1,HE2 | 0.2330 | HE1,H2 | 0.2467 | HE1,HE2 | 0.2098 |
| CZ | 0.6555 | CZ | 0.5064 | CZ | 0.4592 |
| O | -0.7019 | O | -0.5866 | O | -0.5116 |
| P | 1.4716 | P | 1.4755 | S | 1.2909 |
| O1 | -0.9550 | O1P | -0.9113 | O1 | -0.6634 |
| O2 | -0.9550 | O2P | -0.9113 | O2 | -0.6634 |
| O3 | -0.9550 | O1 | -0.7867 | O3 | -0.6634 |
| - | - | H1 | 0.4925 | - | - |
