# Supplementary material for: Hydrogen Bond Strengths in Phosphorylated and Sulfated Amino Acid Residues
Source: PLoS One. 2013 Mar 5;8(3):e57804. doi: 10.1371/journal.pone.0057804 (PMC3589483; doi:10.1371/journal.pone.0057804)
Supplement: Table S2 — Partial Charges for pSer(−2) and pSer(−1). (DOC) [file pone.0057804.s002.doc]

Table S2: Partial Charges for pSer(-2) and pSer(-1).

| *pSer(-2)* | | *pSer(-1)* | |
| --- | --- | --- | --- |
| Atoms | Charges | Atoms | Charges |
| CB | 0.1395 | CB | 0.0747 |
| HB1,HB2 | 0.0283 | HB1,HB2 | 0.0879 |
| O | -0.6333 | O | -0.5470 |
| P | 1.4243 | P | 1.376 |
| O1 | -0.9957 | O1P | -0.8973 |
| O2 | -0.9957 | O2P | -0.8973 |
| O3 | -0.9957 | O1 | -0.7621 |
| - |  | H1 | 0.4772 |
|  |  |  |  |
